# Medical Expert Evidence.—II

**Published:** 1909-05-29

**Authors:** 


### Medico-Legal Points.

### MEDICAL EXPERT EVIDENCE.—II.

(*Continued from p.* 96.)

It is extremely difficult at times to determine who are, in point of competency, experts. As a general rule, it may be said that any practising medical man is competent to express an opinion as an expert on a medical question. Physicians and surgeons are presumed to be acquainted with all matters pertaining to their profession, and to be competent to testify concerning them; and their opinions are admissible in evidence upon questions that are particularly and legitimately embraced in their profession and practice.

In questions of legitimacy and divorce, obstetricians of high standing are consulted on both sides; in questions affecting the sanity of persons, those who have acquired a reputation in the treatment or observation of the insane are selected; in the various obscure injuries resulting from railway accidents, surgeons of repute are summoned as experts to give the results of their experience. There are many of these cases which could not possibly be settled without this collateral aid, the questions at issue not being based on matters of fact occurring within the ordinary range of practice, so much as on an enlarged experience of a particular department. There is, however, a strong public feeling against the admission of the testimony of experts. One writer remarks:—"It is impossible to shut out such evidence altogether, but there is nothing which brings more discredit upon the administration of justice. There is one consequence of its admission which is common to all cases in which it occurs—it is, that no difficulty has ever been found in obtaining any amount of evidence of this description on either side of any point at issue." The cause of this evil is that the solicitors on each side are allowed to search the whole profession until they can find one or more persons ready to adopt their respective views; when once in Court, provided a man can call himself a "doctor," his qualifications and experience sometimes escape a rigid scrutiny. Persons have thrust themselves, or have been thrust into cases as experts without any pretensions to such a title, either by their professional standing or experience. A man may have been engaged for a few years only in the ordinary routine of medical practice, and may have had no special experience in the subject on which an opinion is required. He will be described by his counsel as "a most learned and eminent member of the profession, on whose opinion the jury are as much entitled to rely as on that of the 'highly respectable gentleman' called on the other side."

It is extremely difficult at times, therefore, to determine who are, in point of competency, experts. It is wrong to assume, in relation to any of the physical sciences, that because a man has been a practitioner in it he is equally competent and skilful in all its departments. Generally, to obtain eminence in any science is to limit oneself to some one particular branch of it. Medicine is no exception to this rule. In any event, the law may be considered as simply requiring that the party offered as an expert shall, in the whole view of the case, present evidence of having had the best opportunities for perfecting his knowledge in that particular subject on which his opinion is desired. Beyond this, and as between varying degrees of competency among experts, no other test of qualification can be applied, for no other is, in fact, possible; and since errors in judgment may occur here as well as in any other department of human investigation, there is no means of knowing absolutely whether the witness is qualified to deal, or not, with all the problems which the complexities of any given case may present to him.

As in other cases of opinion evidence, the opinion of a medical expert must be founded on a proper basis of fact, and is not admissible unless it is so founded. It may be based, however, on his acquaintance with the person whose condition is under investigation; and a physician who frequently met a person and was well acquainted with him and frequently attended him has sufficient knowledge to form an intelligent opinion as to whether a given condition resulted from illness or injury. And a medical opinion may also be based upon a medical examination; and though the physician was called and the examination made a long time before or afterwards, he may testify as to the condition of the person at the time when he was called in. Nor is the opinion of a medical expert rendered incompetent by the fact that it was based in part upon the statements of the patient or injured person; though it cannot be based wholly on the patient's statements privately made to the expert. And where the medical expert has not made a personal examination of a patient, the proper practice is to put the question to the witness, citing the supposed facts hypothetically upon which the opinion is wanted. A medical expert, however, cannot give an opinion based on non-existent facts, or facts concerning which he had no knowledge; and he cannot testify to an opinion formed upon information derived from private conversation with another, or upon facts proved by other witnesses where the evidence as to such facts is conflicting, since that would involve a decision of an issue for the jury.

To entitle medical expert testimony as to apprehended consequences of a condition or injury to consideration, the consequences must be such as in the ordinary course of nature are reasonably certain to ensue; consequences which are contingent and speculative or merely possible are incompetent. And the possible effects attending the progress of a disease, and its probable duration, and the results which may ensue to the person afflicted, cannot be given. Nor are medical witnesses competent to testify as to what results are likely to follow from an injury. And a physician cannot speculate upon the difference in effect of an injury to a frail person and an injury to a healthy one; and he is not competent to testify on an issue as to a fractured limb, as to the consequences of a hypothetical second fracture. Expert testimony as to future consequences which are reasonably expected to follow an injury, however, may be given for the purpose of enhancing the damages to be awarded; and a physician may testify as to what results will follow with reasonable certainty from conditions observed by him. Nor does the uncertainty of a medical opinion as to probable future results, founded upon present conditions, prevent it from being competent; and a physician may testify as to the probable length of time that a diseased or injured person will live, though stating that he can only give the probability from the history of other similar cases.

The question as to what certain symptoms indicate is a matter of medical skill, and a particular subject for medical testimony; and a physician may be asked to describe the symptoms which ordinarily and necessarily accompany a specified trouble or injury. And while it may be common knowledge that one coming in personal contact with another infected with a contagious disease, or occupying the same room with him, is exposed to it, whether or not upon a given state of facts a person will be deemed to have been exposed, is a medical question for an expert. And the contagious character of the disease and the length of time its poison retains its vitality are also medical questions presenting subjects for professional opinion; and so are questions as to the tendency of certain conditions to produce sickness, or to make injury more probable. Nor is a physician incompetent to give an opinion as to vital spots and places where it is dangerous to receive an injury or blow, or as to the power of resistance of a skull and as to the force requisite to break it.

The nature and extent of injuries, and the present condition of a person ill or injured, are questions peculiarly within the province of medical skill and science, and always proper to be addressed to an expert. And a physician may give his opinion, based on such conditions and the nature of the injuries, on the question as to the causes from which they arose, and as to whether they were produced by violence or disease, and as to whether such a condition could have existed if described circumstances had taken place, such opinions not being objectionable as speculative. And such opinions may be founded upon a statement of the nature of the injury, and subsequent symptoms and present physical conditions, as testified by others; or upon an hypothesis stating the facts of the case upon which an opinion is desired. And a physician may properly give an opinion as to the cause of a change in condition between two different periods or examinations; or as to whether there might have been an injury which would account for the change, besides the injury in question; and a medical opinion as to whether a described cause would produce existing conditions is competent.

The opinions of medical experts as to the cause of death are always admissible when such cause is involved in doubt and there are no witnesses to the occurrence. And where death might have resulted from different causes, an expert may give his opinion as to how the injuries were inflicted; and as to whether or not they were inflicted before death; or that they could not have been inflicted in a designated way; or as to the length of time since death took place. Such opinions are admissible when founded either on personal knowledge of the facts of the case or upon a statement of the case as testified to by others; and they may be based on the previous condition of the person and the subsequent condition of the body, or upon the condition and position of the body and the pathological condition of its internal organs after death is ascertained by examination.

Medical men may likewise, upon facts testified to either by themselves or by others, give their opinions as to whether a particular blow or injury would be adequate to cause death; or even whether such injury was the actual cause of death in the particular case. And physicians who have examined a person's wounds before and after death may give an opinion as to whether an instrument identified as the one used is such as to produce death when used in the manner described. And a physician may properly testify that he knows no disease which would produce death with certain described symptoms. But a physician cannot be asked whether the testimony and described autopsy are such as to enable a physician to tell the cause of death with any certainty. The question should recite the scope and character of the autopsy.

*(To be concluded.)*

